# Pediatric Oral Cavity Physiologically Based Pharmacokinetic Model to Predict Pharmacokinetics of Mucoadhesive Atropine Gel to Treat Sialorrhea

**DOI:** 10.21203/rs.3.rs-8780503/v1

**Published:** 2026-02-19

**Authors:** Madison Parrot, Nancy Murphy, Joseph E. Rower, Christopher A. Reilly, Danielle Green, Ariel Tarrell, Kevin Watt, Venkata Yellepeddi

**Affiliations:** 1 Division of Clinical Pharmacology, Department of Pediatrics, Spencer Fox Eccles School of Medicine, University of Utah, Salt Lake City, Utah, USA; 2 Department of Molecular Pharmaceutics, Utah Center for Nanomedicine, College of Pharmacy, University of Utah, Salt Lake City, Utah, USA; 3 Division of Complex Care, Department of Pediatrics, University of Utah Health, Salt Lake City, Utah, USA.; 4 Center for Human Toxicology, Department of Pharmacology and Toxicology, University of Utah, Salt Lake City, Utah, USA.; 5 Division of Neonatology, Department of Pediatrics, University of Utah School of Medicine, Salt Lake City, Utah, USA.

**Keywords:** Atropine, pharmacokinetics, sialorrhea, excessive salivation, neurodevelopmental disorders, pediatrics

## Abstract

Sialorrhea is a debilitating condition that significantly impairs quality of life in children with neurodevelopmental disorders, including cerebral palsy and neuromuscular disorders, yet safe and effective pharmacologic treatment options remain limited. Although atropine is widely used for sialorrhea, it is most commonly used off-label as ophthalmic drops administered intraorally; this approach is constrained by poor mucosal retention, frequent dosing, risk of medication errors, and systemic anticholinergic adverse effects. To address these limitations, a novel mucoadhesive atropine oral gel (0.01% weight/weight (w/w)) was developed to enhance intraoral residence time and local absorption while reducing systemic exposure variability. The pharmacokinetics and safety of the atropine gel were evaluated in a Phase I clinical trial in healthy adults, which informed the development and validation of a physiologically based pharmacokinetic (PBPK) model incorporating a mechanistic oral cavity framework. The oral cavity PBPK model accounts for salivary flow, mucosal absorption, swallowing, and saliva-tissue exchange across six compartments, enabling predictions of local and systemic exposure. Pediatric PBPK simulations were scaled from the adult model using Population Estimates for Age-Related Physiology^™^ (PEAR Physiology^™^) to support model-informed dose selection. Simulations identified a minimum pediatric dose range of 0.25 mg/kg/day, with twice-daily dosing to maintain plasma concentrations within the established minimum effective and maximum tolerated concentration range. These results demonstrate the utility of PBPK modeling in model-informed drug development and pediatric dose optimization to support further clinical development of mucoadhesive atropine gel as a safer alternative to off-label atropine eye drops for the management of pediatric sialorrhea.

## Graphical Abstract

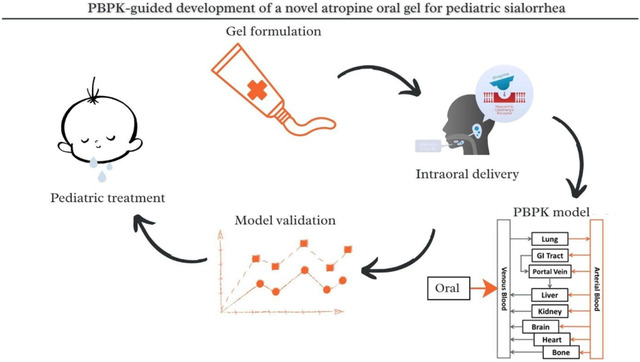

## Introduction

Sialorrhea is excessive salivation that pathologically affects the quality of life of children with neurodevelopmental disorders, including cerebral palsy and dystonia [1–4]. Additionally, for patients of all ages, up to 80% of those with Parkinson’s disease and 30% of those with amyotrophic lateral sclerosis (ALS) experience sialorrhea [5]. Sialorrhea causes respiratory illness that contributes to nearly one-third of hospitalizations in this population [6],[7–19]. Morbidity is high, characterized by chronic aspiration, recurrent pulmonary infections, and progressive lung disease, and is accompanied by a 14-fold increase in mortality risk [20],[21–23,19,24–26]. Despite these problems, management options for sialorrhea are lacking [27,28].

Pharmacological treatment for sialorrhea utilizes anticholinergic agents that block cholinergic muscarinic receptors that are responsible for saliva production, including atropine, glycopyrrolate, benzhexol, scopolamine, and benztropine [3,29,30]. However, currently prescribed systemic use of glycopyrrolate, benzhexol, scopolamine, and benztropine are limited by systemic side effects, including dryness of the mouth, urinary retention, temperature dysregulation, hypohidrosis, and altered central nervous system activity such as blurred vision, drowsiness, confusion, hallucinations, and increased seizures [29,3,31,32]. Off-label use of atropine eye drops to treat sialorrhea is common in institutions across the world and has been proven to be effective [33–41]. However, this off-label treatment is limited by the need for frequent administration due to a lack of retention on the oral mucosa, [33] incidence of dosing and medication errors (for example, accidental ingestion of the entire bottle of eye drops), [42] and systemic side effects such as tachycardia, fever, tremors, and restlessness [43,44]. Collectively, this gap in management constitutes an unmet medical need to develop a safe and effective formulation to treat sialorrhea in children with neurodevelopmental disorders.

To address the limitations associated with the off-label use of atropine eye drops, we developed a mucoadhesive oral gel formulation of atropine at 0.01% weight/weight (w/w). The pharmacokinetics (PK) and safety of the novel atropine oral gel were evaluated in healthy volunteers in a Phase I clinical trial [45]. Intraoral delivery is limited by short residence times; however, the use of mucoadhesive gel can increase both residence time and local absorption. A mucoadhesive atropine gel, designed specifically for oral mucosal application, may reduce dosing errors compared to the off-label use of atropine eye drops. A dedicated formulation with standardized concentration, controlled delivery, and unit-dose administration minimizes variability in delivered dose and reduces the risk of inadvertent high-dose exposure associated with drop-based dosing. Intraoral delivery enables localized delivery not only to three major salivary glands (parotid, submandibular, and sublingual glands) but also to 600–1000 minor salivary glands interspersed across the sublingual region [46].

Few studies have explored intraoral atropine delivery, particularly in the context of children [47]. Establishing appropriate dosing in children is inherently challenging due to developmental changes in physiology, including age-dependent differences in body composition, protein binding, tissue permeability, and drug clearance [48,49]. These maturation-related factors introduce substantial interindividual variability, increasing the risk of both subtherapeutic exposure and dose-limiting toxicity [50]. As a result, pediatric medication use often relies on empirical dose selection or extrapolation from adult data, approaches that may not adequately account for pharmacokinetic differences or route-specific absorption processes [49].

Model-informed drug development (MIDD) provides a rational framework for integrating developmental physiology with drug- and formulation-specific properties to support pediatric dose optimization [51,52]. By mechanistically characterizing absorption, distribution, and early systemic exposure following intraoral administration, MIDD enables quantitative evaluation of how formulation attributes and patient-specific factors influence exposure across age groups. This approach supports the identification of dosing strategies that maximize therapeutic benefit while minimizing adverse effects, particularly in pediatric patients, where drugs such as atropine have a narrow therapeutic window.

Physiologically-based pharmacokinetic (PBPK) models optimize dosing for new formulations in both adult and pediatric populations. PBPK modeling identifies formulations unlikely to succeed via oral cavity delivery due to poor permeability, short residence time, or rapid saliva-mediated clearance. An oral cavity PBPK model is essential to account for salivary partitioning, salivary flow, swallowing, drug absorption, drug dissolution, drug distribution, systemic uptake, the dynamic exchange between saliva and tissue, and diffusivity [53]. The complex physiology of the oral cavity is represented by six distinct compartments: buccal mucosa, gingiva, palate, dorsal tongue, ventral tongue, and the floor of the mouth. This modeling approach improves upon previously used models that assumed direct intraoral absorption into systemic circulation, enabling more precise simulation of local and systemic PK [53].

In the current study, we developed an adult oral cavity PBPK model of atropine and verified its performance using the observed PK data from our atropine gel Phase I study. We then extrapolated the adult atropine oral cavity PBPK model to children. The primary objective of this pediatric PBPK model is to predict systemic exposure after atropine gel administration to ensure safety in pediatric patients. The final pediatric atropine oral cavity PBPK model was used to predict optimal atropine gel dosing in children across the pediatric age spectrum.

## Methods

### Adult physiologically-based pharmacokinetic model development

Modeling and simulation were conducted using GastroPlus^™^ software version 9.9 (Simulation Plus, Lancaster, CA). Intravenous (IV) and oral models were also created and validated with literature data from Schwartz et al. [54] and Mubaslat et al. [55], respectively.

An adult oral cavity PBPK model was developed using Oral Cavity Compartmental Absorption and Transit (OCCAT^™^) module of GastroPlus^®^ software. The physicochemical and physiological input parameters for the oral cavity model are listed in Table 1. The virtual pediatric population, representative of the patient population, ranged in age from 2 to 19 years (n = 100). The OCCAT^™^ model has six physiological compartments: buccal, gingival, palate, top of the tongue, bottom of the tongue, and floor of the mouth [53]. The OCCAT^™^ PBPK model incorporates blood flow, surface area, thickness of epithelial and lamina propria layers, basal saliva volume, saliva production rates (applicable for children with and without excised glands), swallowing pattern (normal vs holding), drug absorption in the oral cavity, tissue diffusion, and system circulation uptake of drug [56]. We replicated the administration pattern used in the Phase I trial, where 50% of the administered dose was deposited in the buccal cavity and 50% was deposited at the base of the tongue.

The primary equations describing the transport of the drug through the layers of the oral cavity in the OCCAT^™^ module are provided as equations 1–4. However, a detailed description of the OCCAT^™^ model can be found in the publication by Xia et al. [53]. Briefly, Equation 1 [53] defines the fraction of drug unbound in the epithelium (*f*_ut_) as the ratio of the unbound drug concentration at the epithelium sublayer 1 to the total drug concentration at the interface, which is also equal to the unbound concentration in saliva. Since the model assumes no nonspecific binding in saliva or buffer, *f*_ut_ is the reciprocal of the saliva/epithelium partition coefficient (*P*).


(1) $$\text{RateIntoSyst}=Q\times R_{\text{bp}}\times \left(C_{\text{u}}^{\left\{\text{lam}\right\}}\times \left(\frac{1}{f_{\text{up}}}\right)-C_{\text{p}}\right)$$

Equation 2 [53] models diffusion across the six hypothetical epithelium sublayers, representing 5% (for layer 1) and evenly divided 95% (for layers 2–6) of total epithelium thickness. Drug diffusion is driven by concentration gradients between adjacent sublayers, where *V*_j_^epi^ is the volume of sublayer *j*, *h*_j_^epi^ is its thickness, and Diff is the drug diffusivity through the epithelium.


(2) $$V_{\text{j}}^{\left\{\text{lam}\right\}}\times \frac{\text{d}C_{\left\{\text{j},\text{t}\right\}}^{\left\{\text{lam}\right\}}}{\text{d}t}=\left(\frac{\text{Diff}}{h_{\text{j}}^{\left\{\text{lam}\right\}}}\right)\times \text{SA}\times \left(C_{\left\{\text{j}-1,\text{u}\right\}}^{\left\{\text{lam}\right\}}-2C_{\left\{\text{j},\text{u}\right\}}^{\left\{\text{lam}\right\}}+C_{\left\{\text{j}+1,\text{u}\right\}}^{\left\{\text{lam}\right\}}\right)-\text{RateIntoSyst}$$

After traversing the epithelium, drug continues diffusing through six evenly spaced lamina propria sublayers. Equation 3 [53] parallels epithelium diffusion but uses lamina propria volumes (V_j_^lam^), sublayer thicknesses (h_j_^lam^), and the unbound and total drug concentrations (*C*_j,t_^lam^ and *C*_j,u_^lam^). It describes movement deeper into tissue toward systemic circulation.


(3) $$V_{\text{j}}^{\text{epi}}*\text{d}C_{\text{j}},\frac{t^{\text{epi}}}{\text{d}t}=\left(\frac{\text{Diff}}{h_{\text{j}}^{\text{epi}}}\right)*\text{SA}*\left(C_{\left\{\text{j}-1,\text{u}\right\}}^{\text{epi}}-2*C_{\left\{\text{j},\text{u}\right\}}^{\text{epi}}+C_{\left\{\text{j}+1,\text{u}\right\}}^{\text{epi}}\right)$$

Drug uptake into the bloodstream occurs via instant equilibrium between the unbound drug in plasma and in each lamina propria layer. The uptake rate depends on lamina propria blood flow (*Q*), the blood/plasma concentration ratio (*R*_bp_), the plasma unbound fraction (*f*_up_), and the unbound drug concentration in lamina propria (*C*_u_^lam^). Equation 4 [53] governs clearance from the lamina propria to systemic circulation.


(4) $$\left(\left(\frac{V_{\text{sal}}}{P}\right)+V_{1}^{\text{epi}}\right)*\frac{\text{d}C_{1,\text{t}}^{\text{epi}}}{\text{d}t}=-\left(\frac{\text{Diff}}{h_{1}^{\text{epi}}}\right)*\text{SA}*\left(C_{\left\{1,\text{u}\right\}}^{\text{epi}}-C_{\left\{2,\text{u}\right\}}^{\text{epi}}\right)$$

Swallowing of the virtual population was set to normal swallowing habits (no holding). Virtual adult participants were administered 1 mL of atropine oral gel containing 0.1 mg of atropine. The observed data originated from the plasma concentration versus time data of the Phase I clinical trial [45]. This data was used to validate the adult oral cavity model.

Parameter sensitivity analysis (PSA) was completed to assess how transmucosal intraoral absorption (*F*_a_IO) is affected by various parameters, including diffusivity, pH, solubility, octanol/water distribution coefficient (logD), fraction unbound in the oral cavity (*f*_ut_), salivary production rates, and the oral partition coefficient.

### Adult physiologically-based pharmacokinetic model evaluation

The details of the steps involved in preparing the atropine oral gel formulation and the data used to evaluate the adult oral cavity PBPK model are presented in our previous publication: Parrot et al. [45]. Atropine oral gel (0.01% w/w) was prepared at the Investigative Drug Services Pharmacy, University of Utah Health, using USP-grade atropine and Carbopol^®^ 974 NF, in accordance with USP 797 guidelines. The gel remained physically, chemically, and microbiologically stable for 30 days at room temperature and under refrigeration. Ten healthy volunteers received a single 1 g dose of the gel, and blood samples were collected at 0, 5, 10, 15, 30, 60 minutes, and 2, 4, 6, 8, and 24 hours, processed, and stored at −80 °C. Atropine plasma concentrations were measured using a validated high-performance liquid chromatography-tandem mass spectrometry (HPLC-MS/MS) assay. The previous single-center, open-label PK study was conducted under the Food and Drug Administration (FDA) investigational new drug (IND) application #155751, IRB approval #00144918, at the Utah Clinical and Translational Science Institute (CTSI), and registered on ClinicalTrials.gov (NCT05164367) [57].

### Extrapolation of the physiologically-based pharmacokinetic model to pediatrics

The pediatric oral cavity PBPK model of atropine was scaled from the adult model using Population Estimates for Age-Related Physiology^™^ (PEAR Physiology^™^). Metabolism of atropine was estimated using ADMET Predictor (v13.0; Simulation Plus Inc., Lancaster, CA). Metabolism is attributed to CYP3A, CYP2D6, and CYP2C19 in the liver microsomes. Specific enzyme kinetics are described in Table 2. Enzyme ontogeny equations are provided in Supplementary Table S1 to account for cytochrome P450 (CYP) maturation [57]. The PBPK model was used to simulate intraoral atropine PK in pediatric populations by incorporating age-dependent physiological parameters and maturation functions. Pediatric simulations utilized PBPK-generated virtual populations stratified by age and weight bands, with demographic distributions informed by data from the National Health and Nutrition Examination Survey (NHANES) [58]. Age-dependent physiological maturation was incorporated, and clinically relevant, weight-adjusted atropine doses were simulated to evaluate pediatric dose-exposure relationships.

### Model verification

Model performance was evaluated using a combination of visual predictive checks (VPCs) and quantitative error metrics to assess agreement between simulated and observed PK data. VPCs included comparison of simulated concentration-time profiles with observed data across relevant dosing conditions. Parameter plausibility checks were also used to verify that the parameters are within physiologically relevant ranges. These diagnostics were used to assess systematic bias and the model’s ability to capture variability.

An average fold error (AFE) and absolute average fold error (AAFE) are used to calculate model fit as follows:

(5) $$\text{AFE}=10^{\frac{1}{n}\sum \text{log}\left(\frac{P_{i}}{o_{i}}\right)}$$

(6) $$\text{AAFE}=10^{\frac{1}{n}\sum \left|\text{log}\left(\frac{P_{i}}{o_{i}}\right)\right|}$$

where *n* is the number of participants and *P*_*i*_ and *O*_*i*_ are the *i*^th^ model-prediction or observation, respectively. AFE is used to assess directional bias (over- and under-prediction), while AAFE quantifies overall prediction accuracy independent of direction. AFE values close to 1 indicate minimal systematic bias, whereas AAFE values closer to 1 reflect higher overall predictive accuracy [59]. Consistent with accepted PBPK modeling practices, model performance was considered acceptable when predictions fell within a 0.5 and 2-fold error bound and demonstrated no systematic deviation across time or exposure metrics [60].

### Pediatric Simulations

For simulations in the pediatric population, three age groups were simulated for each dose: 2–6, 6–12, and 12–19 years (n = 100 per age group). The FDA’s recommendation defined the age groups and weights [50]. The reported maximum toxic concentration (MTC) is 61 ng/mL [61], and the minimum effective concentration (MEC) is 1.46 ng/mL [55]. Each age group was given a once-daily (*quaque die* (QD)) dose of 0.01, 0.05, 0.1, 0.25, or 0.5 mg/kg or twice-daily (*bis in die* (BID)) dose of 0.005, 0.025, 0.05, 0.125, 0.25 mg/kg simulated over a seven-day period. These dose groups were chosen based on clinically-used doses of sublingual atropine to treat sialorrhea in children (0.01–0.49 mg/kg/day) [41].

## Results

### Adult physiologically-based pharmacokinetic model

**Figure 1:**
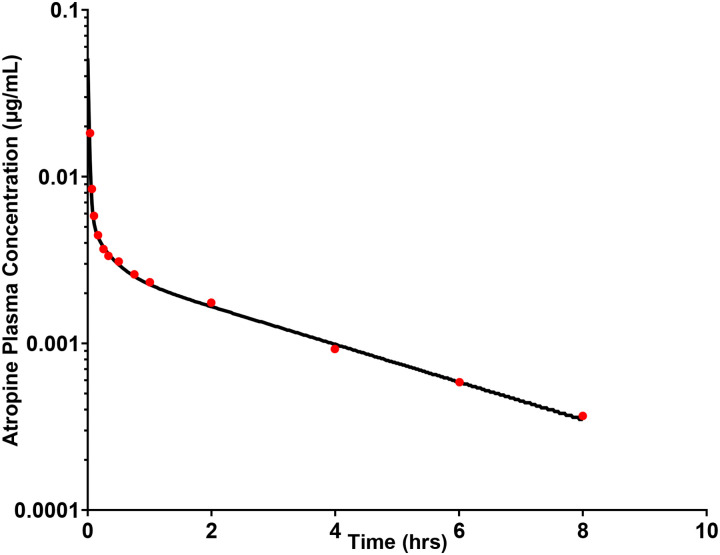
Observed data after IV administration of 1 mg atropine and model-predicted PK. The black line represents the adult IV PBPK model-predicted mean atropine plasma levels in a virtual adult population (N = 100). The red, dark circles represent the observed individual atropine plasma concentrations at each time point from Schwartz et al. (N = 12) [54].

**Figure 2:**
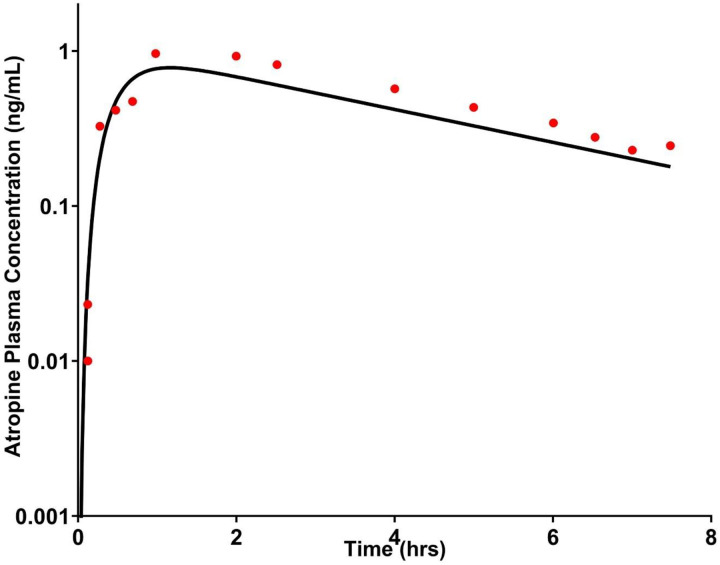
Observed data after oral administration of 0.6 mg atropine and model-predicted PK. The black line represents the adult oral PBPK model-predicted mean atropine plasma levels in a virtual adult population (N = 100). The red, dark circles represent the observed individual atropine plasma concentrations at each time point from Mubaslat et al. (N = 12) [55].

**Figure 3:**
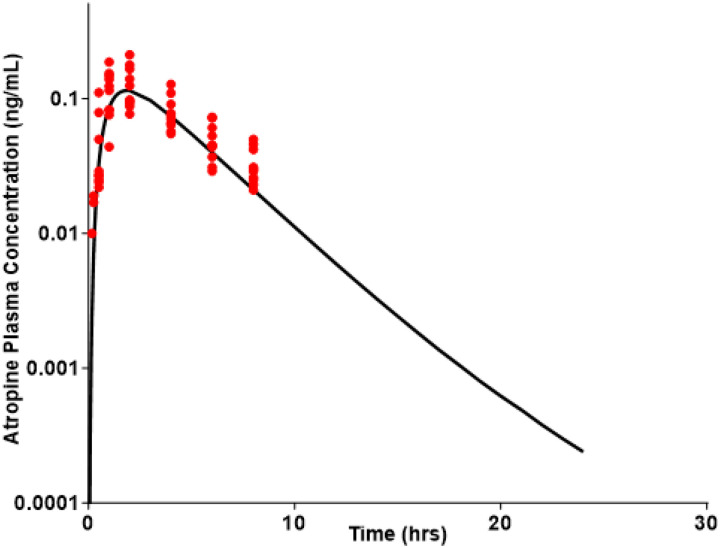
Phase I study observed data after intraoral administration of 0.1 mg atropine gel, comparing PK with adult oral cavity PBPK model-predicted PK. The black line represents the adult oral cavity PBPK model-predicted mean atropine plasma levels in a virtual adult population (N = 100). The red, dark circles represent the observed individual atropine plasma concentrations at each time point from the Phase I PK study in healthy volunteers (N = 10) [45].

The developed PBPK models for atropine accurately captured observed plasma profiles across IV (Figure 1), oral solution (Figure 2), and oral gel (Figure 3) formulations in adults. The adult oral cavity model, validated with Phase I clinical data, demonstrates that 90% of observed oral cavity plasma concentrations fell within the 95% confidence interval of the simulations. The IV, oral, and oral gel atropine models had an AFE and AAFE of 0.937, 1.089, 1.14, 1.44, 0.66, and 1.44, respectively. These results confirmed the successful development and verification of the adult oral cavity PBPK model for atropine gel.

### Parameter sensitivity analysis (PSA)

**Figure 4:**
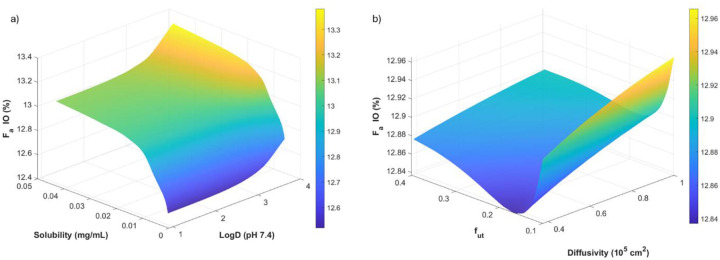
Surface response plots from the results of the parameter sensitivity analysis fraction absorbed *via* intraoral mucosa (*F*_a_IO) by the oral cavity model parameters a) solubility versus logD_pH=7.4_ and b) tissue diffusivity versus *f*_ut_ after a single 0.1 mg dose of atropine oral gel. The color range represents a prediction for *F*_a_IO.

PSA was illustrated on a three-dimensional surface plot (Figure 4). PSA demonstrated that intraoral absorption (*F*_a_IO) was most strongly influenced by physicochemical properties (diffusivity, solubility, and logD), the saliva-epithelium unbound fraction (*f*_ut_), and salivary flow rate. Increases in epithelium permeability and higher diffusivity substantially increased *F*_a_IO, whereas higher salivary production reduced contact time and decreased intraoral absorption. These findings highlight that both formulation properties and oral physiology are major determinants of systemic uptake following oral gel administration.

### Extrapolation of the physiologically-based pharmacokinetic model to pediatrics

**Figure 5:**
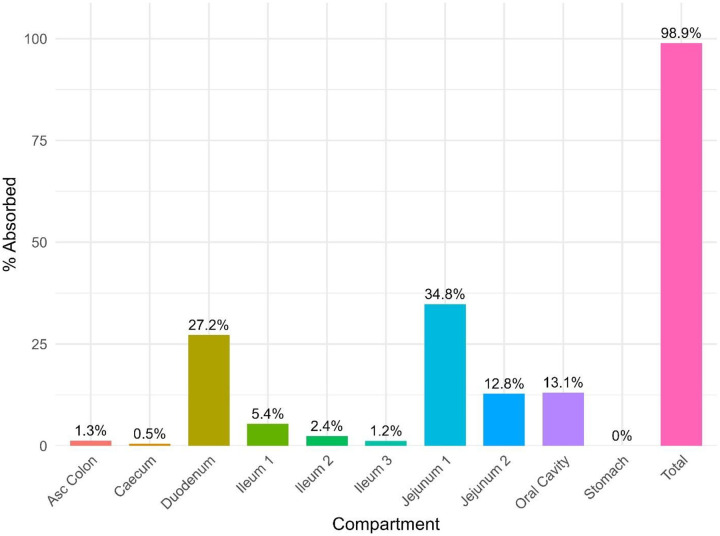
Simulated regional and total fractions absorbed of atropine after 0.1 mg in 1 mL oral gel in adults.

There are essential differences that PEAR Physiology^™^ scaling considers between adults and children, namely, a thinner epithelium, higher blood flow per gram of tissue in children, and higher salivary production rates. Regional absorption fractions are shown in Figure 5. Absorption primarily occurs in the duodenum, jejunum, and oral cavity. Enzyme maturation typically occurs at approximately two years old; therefore, clearance via a metabolic pathway is similar for children and adults.

### Pediatric simulations

**Figure 6:**
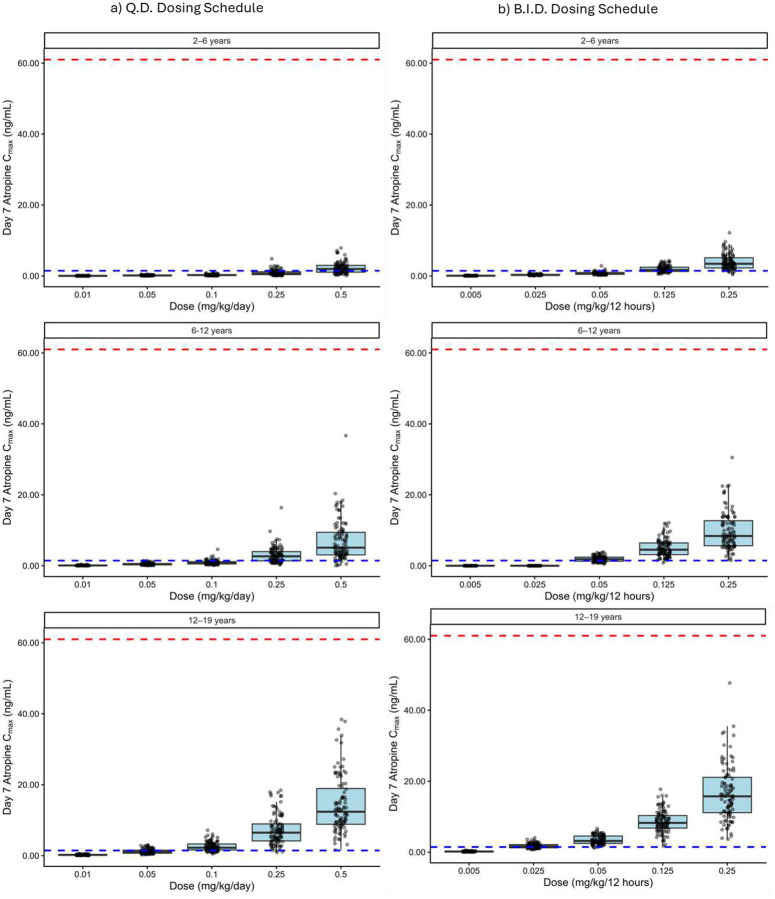
C_max_ on day 7 for age groups 2–6, 6–12, and 12–19 years old after administration of a) 0.01, 0.05, 0.1, 0.25, and 0.5 mg/kg/day, and b) 0.005, 0.025, 0.05, 0.125, and 0.25 mg/kg/12 hours. The minimum effective concentration (MEC) of 1.46 ng/mL is shown with a blue dashed line and the maximum toxic concentration (MTC) of 61 ng/mL is shown with a red dashed line.

Across the 2–19 year virtual population and dose range of 0.01–0.5 mg/day, predicted exposures were consistent with the anticipated age-related differences in oral cavity absorption, tissue diffusion, and salivary clearance. The results of the pediatric population simulations are shown in Figure 6. The results showed that at doses up to 0.5 mg/day of atropine oral gel, plasma atropine levels did not reach the safety target (61 ng/mL). Efficacy simulations indicated that a minimum dose of 0.25 mg/day was required to reach the target plasma concentration of 1.46 ng/mL, associated with approximately a 50% reduction in sialorrhea, with some age dependency in exposure profiles. The pediatric simulations conducted over the 0.01–0.5 mg/day range showed that a minimum of 0.25 mg/kg/day is necessary across all age groups. However, the BID dosing schedule increases the probability of target attainment (PTA) compared to QD, as shown in Table 3. The PTA for 0.5 mg/kg QD in the 2–6, 6–12, and 12–19 years age groups was 64%, 93%, and 100%, respectively. The PTA for 0.25 mg/kg BID in the 2–6, 6–12, and 12–19 year age groups was 93%, 100%, and 100%, respectively.

These qualitative and quantitative diagnostics support the suitability of the PBPK model for simulating intraoral atropine exposure and informing model-informed dosing analyses.

## Discussion

In this study, PBPK modeling played a crucial role in characterizing the impact of key physicochemical properties of atropine, including diffusivity, solubility, and residence time, on its pharmacokinetic performance following intraoral administration in both adult and pediatric populations. By integrating validated IV and oral models as reference standards, we were able to prospectively simulate and compare the PK of atropine gel with conventional formulations.

PBPK models of topical oral therapeutics have been explored, notably an orodispersible film delivery system for risperidone [62] and sublingual tablets of zolpidem [53]. An orodispersible film PBPK model has shown that residence time did not have a significant impact on bioavailability, and the main route of absorption occurs in the upper gastrointestinal tract [62]. The *F*_a_IO of risperidone with an oral residence time of 2 min, 5 min, and 10 min was 7.0%, 11.4%, and 19.5%, respectively [62] and the *F*_a_IO of zolpidem is 18.9% [53], compared to an *F*_a_IO of 13.1% for the atropine. The PBPK model of sublingual zolpidem had a R^2^ > 0.9 for predicted versus observed PK [53]. Xia et al. also explored the relationships between *F*_a_IO and diffusivity, *f*_ut_, logD, and solubility, using the same methodology described in this manuscript. Similarly to atropine oral gel, sublingual zolpidem was also primarily absorbed in the duodenum, jejunum, and oral cavity. While the mucoadhesive gel may increase the oral residence time of atropine compared to sublingual drops, the orodispersible film dissolved within two minutes and showed a similar absorption profile.

Although the rank order of increasing lipophilicity is atropine < zolpidem < risperidone, the fraction absorbed through the oral cavity does not increase proportionally with increasing lipophilicity. While atropine and risperidone are easily ionized in the oral cavity, zolpidem is primarily unionized. In the unionized state, drugs are able absorb through the oral cavity, which offers a possible explanation for zolpidem’s high *F*_a_IO. Zolpidem exhibits the highest intraoral fraction absorbed, despite being less lipophilic than risperidone, while atropine shows a lower FaIO. This non-monotonic relationship reflects the competing roles of lipophilicity, ionization, and solubility in the intraoral environment. Zolpidem possesses a balanced physicochemical profile, with moderate lipophilicity and sufficient aqueous solubility at physiological pH, allowing an adequate dissolved fraction to partition into and permeate the oral mucosa during the short residence time. In contrast, risperidone’s higher lipophilicity is accompanied by lower solubility and greater ionization at oral pH, which limits the amount of dissolved, permeable drug available for absorption despite favorable membrane partitioning. Atropine, although more soluble, is less lipophilic and predominantly ionized, which reduces epithelial partitioning and lowers *F*aIO.

The 3D surface plot evaluating logD (pH 7.4) and solubility illustrates that intraoral absorption is highly constrained, with *F*_a_IO varying by only ~1% absolute (≈12.5–13.4%). This narrow dynamic range is characteristic of the intraoral space, where short residence time, limited surface area, and stratified squamous epithelium restrict the extent of systemic uptake. Within this restricted window, the surface reveals subtle but mechanistically consistent trends. *F*_a_IO, is lowest under low-solubility, high-logD conditions. This pattern reflects two concurrent limitations: reduced dissolution and availability of dissolved species at low solubility, and greater partitioning into the lipophilic epithelial barrier at high logD, which can slow transcellular permeation when the soluble fraction is low.

*F*_a_IO increases modestly toward conditions of higher solubility and moderate logD, indicating that even slight improvements in the available dissolved concentration can produce incremental increases in permeation when residence times are short. The lack of strong dependence on logD suggests that permeation is not strongly lipophilicity-limited in this model and cannot overcome solubility constraints.

*F*_a_IO, is lowest where *f*_ut_ is minimal, even when diffusivity is high. This suggests that binding within the mucosal tissue limits the freely mobile pool that can cross into systemic circulation. Increasing *f*_ut_ yields a consistent but shallow rise in *F*_a_IO, supporting the view that release from tissue binding sites is one of the rate-determining steps. Overall, the surface demonstrates that *F*_a_IO, is most favored when both diffusivity and unbound fraction are high. Still, the total magnitude of improvement is small due to the underlying anatomical limitations of the intraoral space. Diffusivity alone produces modest changes: higher diffusivity yields a slightly higher *F*_a_IO, but the effect is only amplified when *f*_ut_ is also elevated.

This synergy indicates that free, mobile concentration (rather than inherent molecular mobility alone) is the dominant driver. To further formulate the atropine gel to optimize *F*_a_IO, we would focus on the use of the mucoadhesive polymer in the gel (Carbopol^®^) to increase contact time and diffusivity, as manipulation of logP, solubility, or *f*_*ut*_ would require structural changes of atropine, therefore changing its action, based on the results of the PSA. Across all simulations, *F*_a_IO remains tightly clustered around ~13%, underscoring the well-known constraints of intraoral drug delivery such as short contact time, limited surface area, and a barrier optimized for protection rather than absorption. Physicochemical properties such as logD, solubility, diffusivity, and *f*_ut_ exert only secondary effects, producing modest shifts consistent with known mucosal transport mechanisms. These findings collectively suggest that formulation strategies, such as prolonging residence time, enhancing intraoral permeability, or optimizing dissolution kinetics, are far more likely to meaningfully impact intraoral absorption than manipulation of the small-molecule physicochemical properties.

The application of PBPK modeling during preformulation provides a robust, mechanistic tool to inform formulation strategy and compound selection before *in vivo* studies. Notably, PBPK modelling enabled early prediction of whether intraoral administration would provide sufficient systemic absorption, highlighting the role of residence time and mucosal diffusivity as critical determinants of intraoral bioavailability. Sensitivity analyses further elucidated the interplay between solubility, diffusivity, and tissue partitioning, offering guidance on acceptable property ranges to optimize the formulation. In pediatric simulations, anatomical and physiological differences in oral cavity surface area, saliva flow, and epithelium thickness can modulate absorption dynamics, underscoring the value of PBPK models in informing pediatric formulation design where clinical data are often limited.

The half-life of the atropine oral gel is 3.02 hr^−1^ [45]. Drugs are 97% eliminated in five half-lives, [63] therefore, after 15 hours, the atropine gel is nearly eliminated. By dosing every 12 hours, the efficacy of atropine will be increased by maintaining its systemic level above zero and closer to the MEC. Dramatic oscillation of atropine levels in and out of the therapeutic window leads to worse symptom control and breakthrough symptoms. Maintenance of atropine levels above the MEC concentration allows accumulation and the attainment of steady-state levels. BID dosing will enable clean elimination-phase data (without approaching the lower limits of quantification) in the future Phase II trial. In fact, regulatory guidance recommends avoiding carryover in crossover trials to prevent the first period from interfering with the second period and introducing bias [64,65]. BID dosing is recommended for the atropine oral gel. Furthermore, twice-daily dosing is reasonable for children, as it is not too frequent and thus limits a possible propensity for non-compliance.

There are limitations to this work. Firstly, the pediatric model predictions have not yet been validated against *in vivo* or clinical datasets specific to intraoral atropine gel delivery, as these data are not yet available. The adult oral model is derived from healthy adults, not those with underlying conditions. The parameters explored via PSA may vary dynamically with hydration, mucosal thickness, local blood flow, and patient disease states (e.g. dysphagia). As a result, the predicted *F*_a_IO may not fully capture inter-individual variability in clinical settings. Additionally, salivary flow was modeled with fixed values, though it may be substantially higher during acute physiological stress in persons with sialorrhea.

Overall, these findings demonstrate how PBPK modeling can support preformulation decisions by identifying promising delivery approaches, informing formulation attributes such as mucoadhesive strength or particle size, and refining dose selection. This translational modeling approach reduces the need for extensive empirical formulation screening. It enhances the efficiency of formulation development, particularly for alternative routes like intraoral delivery, where residence time and local physiology critically influence absorption.

## Conclusion

Mucoadhesive atropine gel has an acceptable safety profile and favorable PK in healthy adults, as established in a Phase I clinical trial, and these data supported the successful development and validation of an adult oral cavity PBPK model [45]. A pediatric PBPK model was subsequently scaled from adults using PEAR^™^ physiology to inform dose selection, predicting a pediatric dose range of 0.1–0.5 mg/kg/day, with twice-daily dosing recommended. The mucoadhesive gel formulation may offer a clinically advantageous alternative to the off-label use of atropine eye drops for managing sialorrhea. Together, these model-informed insights support a MIDD approach to pediatric development by reducing reliance on empirical trial-and-error experimentation and informing future Phase II efficacy study design in a sensitive population.

## Supplementary Material

1

Supplementary Files

This is a list of supplementary files associated with this preprint. Click to download.

- S1.docx

## Figures and Tables

**Table 1:** Parameter values used in the PBPK model.

Parameter	Units	Value	Source
LogD		1.83	[67]
pKa		9.32	ADMET Predictor 13.0
Peff	×10^−4^ cm^2^/second	2.24	ADMET Predictor 13.0
Solubility	mg/mL	6.72	ADMET Predictor 13.0
Molecular weight	(Da)	289.38	[68]
Unbound fraction in plasma	%	81.57	ADMET Predictor 13.0
Unbound fraction in oral tissue	%	53	GastroPlus parameter estimate
First-pass gut extraction	%	10	Estimation from bioavailability
Renal clearance	L/hour	77	GastroPlus parameter estimate
Hepatic clearance	L/hour	144	Estimation from clinical trial data [45]
Oral mucosa diffusivity	×10^−6^ cm^2^/s	0.14	GastroPlus parameter estimate
Buccal blood flow	(mL/min/100 g tissue)	22.78	[69,70]
Buccal surface area	cm^2^	50.2	[71–73]
Buccal epithelium thickness	μm	418.8	[74–81]
Buccal lamina propria thickness	μm	500	[74]
Buccal pH		6.3	[82]
Tongue-bottom blood flow	(mL/min/100 g tissue)	15.84	[70,69]
Tongue-bottom surface area	cm^2^	13.3	[71–73]
Tongue-bottom epithelium thickness	μm	235	[74–81]
Tongue-bottom lamina propria thickness	μm	250	[74]
Tongue-bottom pH		6.5	[82]

**Table 2:** Enzyme kinetics as predicted by ADMET Predictor (v13.0; Simulation Plus Inc., Lancaster, CA).

Enzyme	Parameter	Value
CYP3A	K_m_ (μM)	31.183
	V_max_ (mg/second)	0.2835
CYP2D6	K_m_ (μM)	5.278
	V_max_ (mg/second)	0.00466
CYP2C19	K_m_ (μM)	6.7428
	V_max_ (mg/second)	0.11324

**Table 3:** Probability of target attainment (PTA) of each dose in each age group (2–6, 6–12, and 12–19 years old) for *quaque die* (QD) and *bis in die* (BID) dosing regimens.

Dosing Regimen		PTA (%)	
	2–6 years old	6–12 years old	12–19 years old
0.01 mg/kg QD	0	0	0
0.05 mg/kg QD	0	0	31
0.1 mg/kg QD	0	16	79
0.25 mg/kg QD	19	70	97
0.5 mg/kg QD	64	93	100
0.005 mg/kg BID	0	0	0
0.025 mg/kg BID	0	0	60
0.05 mg/kg BID	4	67	97
0.125 mg/kg BID	61	99	100
0.25 mg/kg BID	93	100	100
