# Supplementary material for: Pediatric Oral Cavity Physiologically Based Pharmacokinetic Model to Predict Pharmacokinetics of Mucoadhesive Atropine Gel to Treat Sialorrhea
Source: Res Sq. 2026 Feb 19:rs.3.rs-8780503. Preprint. [Version 1] doi: 10.21203/rs.3.rs-8780503/v1 (PMC12934994; doi:10.21203/rs.3.rs-8780503/v1)
Supplement: 1 [file NIHPPRS8780503V1-supplement-1.pdf]

Supplementary materials

Supplementary Table S*1* Hyperbolic functions of the fraction of CYP abundance [57].

**Enzyme**	**Hyperbolic function**
CYP3A	$\frac{0.639 * Age}{2.36 + Age} + 0.42$
CYP2D6	$\frac{1.01 * Age}{0.101 + Age} + 0.036$
CYP2D18/19	$\frac{0.857 * Age}{0.99 + Age} + 0.23$
